# Numerical study of interfacial solitary waves propagating under an elastic sheet

**DOI:** 10.1098/rspa.2014.0111

**Published:** 2014-08-08

**Authors:** Zhan Wang, Emilian I. Părău, Paul A. Milewski, Jean-Marc Vanden-Broeck

**Affiliations:** 1 Department of Mathematics, University College London, Gower Street, London WC1E 6BT, UK; 2 School of Mathematics, University of East Anglia, Norwich NR4 7TJ, UK; 3 Department of Mathematical Sciences, University of Bath, Bath BA2 7AY, UK

**Keywords:** gravity–flexural, interfacial wave, solitary wave, generalized solitary wave

## Abstract

Steady solitary and generalized solitary waves of a two-fluid problem where the upper layer is under a flexible elastic sheet are considered as a model for internal waves under an ice-covered ocean. The fluid consists of two layers of constant densities, separated by an interface. The elastic sheet resists bending forces and is mathematically described by a fully nonlinear thin shell model. Fully localized solitary waves are computed via a boundary integral method. Progression along the various branches of solutions shows that barotropic (i.e. surface modes) wave-packet solitary wave branches end with the free surface approaching the interface. On the other hand, the limiting configurations of long baroclinic (i.e. internal) solitary waves are characterized by an infinite broadening in the horizontal direction. Baroclinic wave-packet modes also exist for a large range of amplitudes and generalized solitary waves are computed in a case of a long internal mode in resonance with surface modes. In contrast to the pure gravity case (i.e without an elastic cover), these generalized solitary waves exhibit new Wilton-ripple-like periodic trains in the far field.

## 1. Introduction

Internal gravity waves are ubiquitous in the ocean owing to density stratification arising from salinity and temperature variations. Waves are often generated by tidal or other currents flowing over topography in the stratified ocean. Internal waves play an important role in transferring heat, energy and momentum in both horizontal and vertical directions and, owing to relatively small density differences in the fluid, are much lower than surface waves at the interface between water and air. Internal *interfacial* waves arise in situations when the stratification has sharp density variations such as at the lower boundary of the surface mixed layer of the ocean, at the interface between fresh and sea water at the mouths of rivers, or in exchange flow situations such as in the Strait of Gibraltar. A simple mathematical idealization for studying internal interfacial waves is the wave propagation on the sharp density discontinuity between two immiscible fluids. The ‘rigid lid’ approximation is commonly used for this two-layer system, which means the top of the upper layer is bounded by a horizontal rigid wall. However, the well-known ‘dead-water’ phenomenon, first reported by Fridtjöf Nansen whose ship experienced an unexpected strong resistance when he was sailing on calm seas in 1893, implies that the interaction between the upper free surface and the internal interface in a density-stratified fluid may be important. Ekman [1] produced a series of elegant laboratory experiments on the dead-water problem, and explained that the force exerted by the ship to propel itself forward was instead translated into the generation of long internal waves which caused an apparent drag on the ship.

In the polar region, floating sea ice is subjected to wave motion and can both generate and respond to the internal waves. The wind-driven small ice pack is a source of internal wave generation in the form of pressure ridge keels (see [2] and the references therein). A heavy mass moving on a large floating ice sheet with small velocity can excite internal waves if the underlying water is stratified, and may experience an anomalous drag analogous to the dead-water phenomenon (see [3] for details). Furthermore, the ice cover, which can suppress the small-scale surface wave noise such as wave turbulence and wind ripples, can provide a surface signature for internal waves. Czipott *et al.* [4] reported coherent measurements of ice tilt forced by internal waves in the Arctic Ocean. This paper is motivated by the physical significance of the coupling between internal waves and sea-ice cover in the polar region. This work is devoted to the numerical study of the permanent coherent structures—solitary waves and generalized solitary waves—in an idealization for the physical problem, namely a two-layer fluid system with a density interface and covered by a flexible elastic sheet that resists flexural motion.

Interfacial waves have been intensively investigated over the past several decades in modelling, theory and numerics. Under the ‘rigid lid’ approximation and the long wave assumption (the thickness of one or both layers are small compared with the characteristic wavelength), different model equations can be derived to study two-layer interfacial gravity waves. On the numerical side, for travelling waves, Meiron & Saffman [5] and Turner & Vanden-Broeck [6] first computed fully nonlinear two-dimensional gravity interfacial waves, including both periodic and solitary waves.

More recently, some three-dimensional studies have included either interfacial effects or flexural surface effects. For example, fully localized three-dimensional interfacial gravity–capillary solitary waves were found by Părău *et al.* [7]. Three-dimensional surface flexural gravity waves are considered, in the presence of a pressure forcing in Părău & Vanden-Broeck [8], in the asymptotic limit of wave-packet dynamics in Milewski & Wang [9] and localized solitary waves are described in Wang *et al.* [10].

There are relatively fewer studies on the interfacial waves with free surface. For the pure gravity waves, Moni & King [11] computed barotropic solitary waves—waves in which both the interface and free surface are elevation waves—using the irrotational Euler equation. Michallet & Dias [12] and Părău & Dias [13] computed solitary waves with non-decaying periodic tails which are usually called generalized solitary waves. An extensive numerical study on interfacial solitary waves with free surface was recently carried out by Woolfenden & Părău [14] when the capillary effect was included either on the free surface of the upper layer or on both the top surface and the interface. Theoretical work by Iooss [15] has considered the spectrum of the linearized operator near equilibrium when the lower layer is infinitely deep. A number of model equations have been introduced and investigated in the spirit of long wave or Boussinesq approximation, including for example Dias & Il'chev [16], Fochesato *et al.* [17] and Craig *et al.* [18].

In this paper, we numerically study steady interfacial gravity waves in a two-layer fluid system with an upper free surface consisting of a flexible elastic sheet modelling the floating ice cover. For the sheet, we use a fully nonlinear elasticity model based on the special Cosserat theory of hyperelastic shells proposed by Toland [19] for the study of flexural–gravity waves. Therefore, there are two restoring forces across the elastic sheet: gravity and an elastic bending force which appears as a pressure jump in the Bernoulli equation at the top surface.

The paper is structured as follows. In §2, we briefly present the mathematical formulation of the full problem and its linear behaviour. In §3, we describe the boundary integral method and the series truncation method, and thereafter present numerical results for steady symmetric waves, including both solitary waves and generalized solitary waves. In the conclusion, we summarize our new findings and discuss possible extensions to the work.

## 2. Formulation

### (a) Mathematical formulation

**Figure 1.**
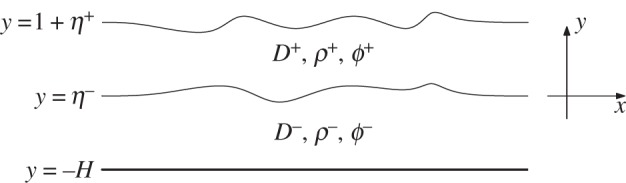
Schematic description of the two-layer fluid geometry after non-dimensionalization.

Consider a two-dimensional incompressible, inviscid fluid bounded below by a rigid horizontal wall and above by an elastic sheet. For the sake of simplicity, we assume that the fluid is composed of two immiscible layers separated by a sharp interface. The density in each layer is assumed to be constant: a lighter fluid with density *ρ*^+^ lies on a heavier fluid with density *ρ*^−^ (figure 1). Cartesian coordinates (*x*,*y*) are introduced such that the *y*-axis points vertically upwards, and the *x*-axis lies in the line of the undisturbed interface. In each layer, the flow is irrotational, and the deformations of the elastic cover and of the interface between the two layers propagate at a constant velocity *c*. Therefore, we can choose a frame of reference moving with velocity *c* and reduce the problem to a steady one. Let *h*^+^ and *h*^−^ be the undisturbed depths of the upper and lower layers, respectively, and denote the equations of the perturbations of the interface and of the upper free surface by *y*=*η*^−^(*x*) and *y*=*η*^+^(*x*)+*h*^+^.

A natural non-dimensionalization of the whole system involves the depth of the upper layer *h*^+^ and the speed *c* as reference quantities. Therefore, we define the following non- dimensional numbers:

2.1 $$H=\frac{h^{-}}{h^{+}},\;R=\frac{\rho^{+}}{\rho^{-}}<1\;\mathrm{and}\;F=\frac{c}{\sqrt{gh^{+}}},$$

where *g* is the acceleration of gravity and *F* is the Froude number. For the sake of simplicity, we continue denoting by *η*^±^ the disturbance of the top free surface and the interface after rescaling. The velocity potentials *ϕ*^+^ in the upper layer and *ϕ*^−^ in the lower layer are both governed by the Laplace equations

2.2 $$\phi_{xx}^{-}+\phi_{yy}^{-}=0,\;-H<y<\eta^{-}(x)$$

and

2.3 $$\phi_{xx}^{+}+\phi_{yy}^{+}=0,\;\eta^{-}(x)<y<1+\eta^{+}(x).$$

At the rigid bottom *y*=−*H*, the slip boundary condition reads

2.4 $$\phi_{y}^{-}=0.$$

There are two kinematic boundary conditions and a pressure balance condition on the interface *y*=*η*^−^(*x*), which are given by

2.5 $$\phi_{y}^{-}=\phi_{x}^{-}\eta_{x}^{-},\;\phi_{y}^{+}=\phi_{x}^{+}\eta_{x}^{-}$$

and

2.6 $$\frac{F^{2}}{2}[|\nabla \phi^{-}{|}^{2}-R|\nabla \phi^{+}{|}^{2}-(1-R)]+(1-R)\eta^{-}=B^{-},$$

where *B*^−^ is a Bernoulli constant.

Finally for the top free surface, the two restoring forces are gravity and the flexural elasticity of the elastic cover. We neglect the inertia and the thickness of the plate. These two effects can be easily incorporated into the system for steady problems (see [20] for the expression of the inertia and [21] for how to model the system with the thickness of the plate). The pressure jump exerted by the ice sheet owing to flexing—even with these approximations—has been modelled with a variety of methods (see [9] for a comparison). In this paper, we use the Cosserat model introduced by Toland for this problem (see [19] for the detailed derivation). In contrast to the Kirchhoff–Love model, this model supports both elevation and depression solitary waves. Therefore, the kinematic and dynamic boundary conditions at the free surface *y*=1+*η*^+^(*x*) take the form

2.7 $$\phi_{y}^{+}=\phi_{x}^{+}\eta_{x}^{+}$$

and

2.8 $$\frac{F^{2}}{2}[|\nabla \phi^{+}{|}^{2}-1]+\eta^{+}+E_{b}\left(\partial_{ss}\kappa +\frac{\kappa^{3}}{2}\right)=B^{+}.$$

Here, *B*^+^ is another Bernoulli constant, *κ* is the curvature of the free surface deformation, *s* is the arc-length parameter and

$$E_{b}=\frac{Eh^{3}}{12(1-\nu^{2})\rho^{+}g{\left(h^{+}\right)}^{4}}$$

is the dimensionless flexural rigidity of the ice sheet, where *E* is the Young modulus, *ν* is the Poisson ratio and *h* is the thickness of the elastic sheet.

### (b) Linear theory

We linearize the whole system (2.2)–(2.8) around the trivial uniform stream solution: *B*^−^=*B*^+^=0, *ϕ*^−^=*ϕ*^+^=*x* and *η*^−^=*η*^+^=0, i.e. for arbitrary positive wavenumber *k*, we substitute the regular expansion ansatz

$$\begin{array}{rl} \phi^{-} & =x+\epsilon {\hat{\phi }}^{-}\cosh[k(H+y)]\sin(kx)\\ \phi^{+} & =x+\epsilon [{\hat{\phi }}_{1}^{+}\cosh(ky)+{\hat{\phi }}_{2}^{+}\sinh(ky)]\sin(kx)\\ \eta^{-} & =\epsilon {\hat{\eta }}^{-}\cos(kx)\\ \eta^{+} & =\epsilon {\hat{\eta }}^{+}\cos(kx) \end{array}$$

into the equations (2.5)–(2.8) and drop the terms of *O*(*ϵ*^2^) and higher. We then obtain a homogeneous linear algebraic system for ${\hat{\phi }}^{-}$, ${\hat{\phi }}_{1}^{+}$, ${\hat{\phi }}_{2}^{+}$, ${\hat{\eta }}^{-}$ and ${\hat{\eta }}^{+}$. By requiring the solution for this system to be non-trivial, we obtain, after some algebra, the linear dispersion relation in the form of

2.9 $${\left(F^{\pm }\right)}^{2}=\frac{b(k)\pm \sqrt{b^{2}(k)-4a(k)c(k)}}{2ka(k)}$$

with

2.10 $$\begin{array}{r} a(k)=1+R\tanh k\tanh(kH), \end{array}$$

2.11 $$\begin{array}{r} b(k)=\tanh k+\tanh(kH)+E_{b}k^{4}[\tanh k+R\tanh(kH)] \end{array}$$

2.12 $$\begin{array}{r} \text{and}\;c(k)=(1-R)(1+E_{b}k^{4})\tanh k\tanh(kH). \end{array}$$

There are two distinct Froude numbers *F*^+^ and *F*^−^ in the dispersion relation which are referred to as the external mode (also called fast mode) and internal mode (also called slow mode), respectively. It is obvious that ${\left(F^{+}\right)}^{2}\to \infty$ and ${\left(F^{-}\right)}^{2}\to 0$ as k→∞.

We first consider the external and internal modes in the long wave regime. Then, the wavenumber *k* is close to 0, and these two modes can be expanded as

2.13 $${\left(F^{+}\right)}^{2}=\frac{1}{2}(1+H+A_{1})-\alpha^{+}k^{2}+O(k^{4})$$

and

2.14 $${\left(F^{-}\right)}^{2}=\frac{1}{2}(1+H-A_{1})-\alpha^{-}k^{2}+O(k^{4}),$$

where

2.15 $$\begin{array}{r} \alpha^{+}=\frac{1}{2}\left[RH(1+H+A_{1})+\frac{1+H^{3}}{3}+\frac{A_{2}}{A_{1}}\right], \end{array}$$

2.16 $$\begin{array}{r} \alpha^{-}=\frac{1}{2}\left[RH(1+H-A_{1})+\frac{1+H^{3}}{3}-\frac{A_{2}}{A_{1}}\right], \end{array}$$

2.17 $$\begin{array}{r} A_{1}=\sqrt{{\left(1+H\right)}^{2}-4H(1-R)} \end{array}$$

2.18 $$\begin{array}{r} \text{and}\;A_{2}=\frac{\left(H-1)(H^{3}-1\right)}{3}+2RH\left[\frac{1+H^{2}}{3}+(1-R)H\right]. \end{array}$$

**Figure 2.**
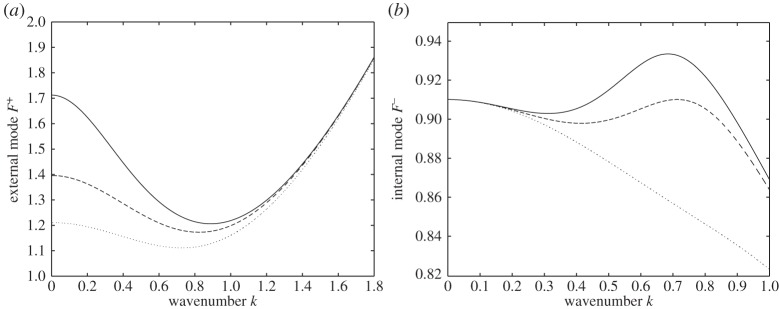
(*a*) External (fast) mode for *R*=0.9,*E*_b_=0.5. Solid line: *H*=2; dashed line: *H*=1; dotted line: *H*=0.5. (*b*) Internal (slow) mode for *R*=0.1, *H*=2. Solid line: *E*_b_=2.5; dashed line: *E*_b_=1.4755; dotted line: *E*_b_=0.5.

It follows that *α*^+^ is positive definite for arbitrary 0<*R*<1, but the sign of *α*^−^ is negotiable and depends on the choices of *H* and *R* (it is not difficult to verify this by considering the case of large *H*). This fact implies that the minimum of the external mode always occurs at a non-zero wavenumber (typical dispersion curves for *F*^+^ are presented in figure 2*a*). In figure 2*b,* we present three dispersion curves for internal modes. It shows that the maximum of the internal mode can occur at a non-zero wavenumber or at zero wavenumber depending on the choices of the parameters *H*, *R* and *E*_b_.

Another terminology also commonly used in the theory of multi-layer fluids is that of ‘barotropic’ and ‘baroclinic’ modes. In the case of a two-layer fluid with a free surface, the barotropic mode is the one whose waves on the free surface and on the interface move in phase with each other (i.e. both interfaces are elevated or depressed simultaneously). On the other hand, if the two waves have opposite phases, they are denoted as the baroclinic mode. In linear theory, the phase relationship between the top free surface and the interface is determined by the sign of ${\hat{\eta }}^{+}/{\hat{\eta }}^{-}$. This can be expressed as

2.19 $$\frac{{\hat{\eta }}^{+}}{{\hat{\eta }}^{-}}=\cosh(k)-\frac{\left[1-R-k\coth(kH)F^{2}]\sinh(k\right)}{kRF^{2}}.$$

We now consider this ratio in the long wave limit. This gives

2.20 $$\lim_{k\to 0^{+}}{\frac{{\hat{\eta }}^{+}}{{\hat{\eta }}^{-}}|}_{F=F^{\pm }}=\frac{1}{RH{\left(F^{\pm }\right)}^{2}}\left[\frac{2(1+RH)H(1-R)}{(1+H)\mp \sqrt{{\left(1+H\right)}^{2}-4H(1-R)}}-H(1-R)\right].$$

By using (2.13), (2.14), (2.17) and (2.18), it is easy to show that the right-hand side of (2.20) is positive for *F*^+^ and is negative for *F*^−^. It follows that the internal modes are baroclinic and the external modes are barotropic in the long wave regime.

**Figure 3.**
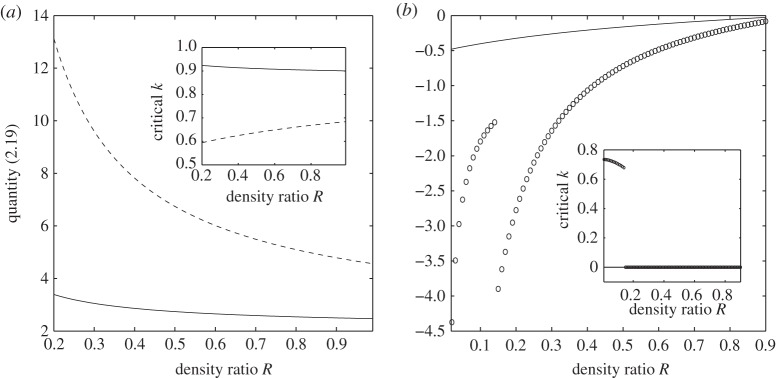
The nature of the modes at the extrema (described in the text) of *F*^±^(*k*). (*a*) Fast modes *F*^+^ with *E*_b_=0.5. Solid line: *H*=3; dashed line: $H=\frac{1}{3}$. The large graph shows that the modes are barotropic, whereas the inset shows the extrema occur at finite *k*. (*b*) Slow modes *F*^−^. Solid line: $H=\frac{1}{3},\;E_{b}=0.1$; circles: *H*=3,*E*_b_=2. The large graph shows that the modes are baroclinic, whereas the inset shows the extrema switch between *k*=0 and finite *k*.

We now consider the regime for wavenumbers away from *k*=0. In terms of localized travelling waves, the most interesting points on the dispersion curves are the global minimum of the external mode and the global maximum of the internal mode. Gap solitary waves are likely to be found between these two extrema, otherwise they would resonate with linear periodic waves, resulting generically in non-decaying tails in the far field. The value of *k* at which these extrema are attained is called ‘critical *k*’. Figure 3 illustrates the behaviour of the critical *k* and the types of waves that occur (baroclinic or barotropic) for some values of the physical parameters. By fixing *E*_b_=0.5 and varying *R* between 0.2 and 1, figure 3*a* shows that the minimum of *F*^+^ is always attained at non-zero wavenumber (see the inset in figure 3*a* where we plot the critical value of *k* versus *R*), and that the external modes are barotropic for both *H*=3 (solid line) and $H=\frac{1}{3}$ (dashed line). Figure 3*b* considers the maximum of *F*^−^. For a small coefficient of flexural rigidity, it occurs at zero wavenumber, and the quantity of (2.19) is negative-definite from (2.20) (see the solid line in figure 3*b* with *E*_b_=0.1 and $H=\frac{1}{3}$). For another set of parameters (*E*_b_=2 and *H*=3), there is a jump at *R*≈0.15 where the maximum of *F*^−^ transits from a non-zero wavenumber to zero wavenumber as the density ratio increases, whereas the modes at these maxima are still of baroclinic type (see the circles in figure 3*b*).

## 3. Numerical results

### (a) Solitary waves

#### (i) Boundary integral method

Here, we describe the numerical method for the fully localized travelling wave solution of the nonlinear problem defined by (2.2)–(2.8). The hodograph transform is used in the numerical scheme, and the auxiliary function (*f* below) is introduced to connect the potential function change across the interface. This method was first used by Vanden-Broeck [22], and later extended by Turner & Vanden-Broeck [6], Moni & King [11], Laget & Dias [23], Woolfenden & Părău [14] and others.

The complex potential in each layer is represented by

3.1 $$w^{\pm }(z)=\phi^{\pm }(z)+i\psi (z),$$

where *ϕ* is the potential, *ψ* is the stream function and *z*=*x*+*iy*. We shall use a hodograph transformation exchanging the dependent and independent variables. Now, *ϕ*^±^ are the independent variables along the interface and free surface. Without loss of generality, we choose *x*=0 at *ϕ*^+^=*ϕ*^−^=0 and write *ϕ*^+^=*f*(*ϕ*^−^). On the interface, we choose *ψ*=0. It follows from the choice of the dimensionless variables that *ψ*=−*H* on the rigid bottom and *ψ*=1 on the free surface. We use *ϕ*^−^ as the main variable. Therefore, the profiles of the interface and of the free surface are parametrized by *ξ*^−^(*ϕ*^−^), *ξ*^+^(*ϕ*^−^), *η*^−^(*ϕ*^−^) and 1+*η*^+^(*ϕ*^−^), respectively. The lower layer is mapped into the infinite strip −*H*<*ψ*<0 of the *ϕ*^−^+*iψ* plane. Using the method of images, the kinematic boundary condition on the horizontal bottom is satisfied by reflecting the lower layer into the bottom. This resulting lower layer is mapped into the infinite strip −2*H*<*ψ*<0 of the *ϕ*^−^+*iψ* plane. We suppress the superscript in *ϕ*^−^ for the sake of simplicity. Applying the Cauchy integral formula to the lower fluid, we obtain

3.2 $$\xi_{\phi }^{-}(\phi_{m})-1=\frac{1}{\pi }\left[\int_{-\infty }^{\infty }\frac{2H(\xi_{\phi }^{-}-1)-(\phi -\phi_{m})\eta_{\phi }^{-}}{{\left(\phi -\phi_{m}\right)}^{2}+4H^{2}}\;d\phi -\int_{-\infty }^{\infty }\frac{\eta_{\phi }^{-}}{\phi -\phi_{m}}\;d\phi \right].$$

For the upper layer, we need to apply the Cauchy integral formula to both the free surface and the interface. This yields the following two equations:

3.3 $$\frac{\xi_{\phi }^{-}}{f_{\phi }}(\phi_{m})-1=\frac{1}{\pi }\left[\int_{-\infty }^{\infty }\frac{(\xi_{\phi }^{+}-f_{\phi })-[\;f(\phi )-f(\phi_{m})]\eta_{\phi }^{+}}{{\left[\;f(\phi )-f(\phi_{m})\right]}^{2}+1}\;d\phi +\int_{-\infty }^{\infty }\frac{\eta_{\phi }^{-}}{f(\phi )-f(\phi_{m})}\;d\phi \right]$$

and

3.4 $$\frac{\xi_{\phi }^{+}}{f_{\phi }}(\phi_{m})-1=\frac{1}{\pi }\left[\int_{-\infty }^{\infty }\frac{(\xi_{\phi }^{-}-f_{\phi })+[\;f(\phi )-f(\phi_{m})]\eta_{\phi }^{-}}{{\left[\;f(\phi )-f(\phi_{m})\right]}^{2}+1}\;d\phi -\int_{-\infty }^{\infty }\frac{\eta_{\phi }^{+}}{f(\phi )-f(\phi_{m})}\;d\phi \right].$$

The dynamic boundary conditions on the top free surface and the interface are used to close the system. In the transformed plane, the dynamic boundary condition takes the form

3.5 $$\frac{F^{2}}{2}\left(\frac{f_{\phi }^{2}}{J^{+}}-1\right)+\eta^{+}+\frac{E_{b}}{2}\left[\frac{\kappa_{\phi \phi }}{J^{+}}+{\left(\frac{\kappa_{\phi }}{J^{+}}\right)}_{\phi }+\kappa^{3}\right]=0,$$

where

3.6 $$J^{+}={\left(\xi_{\phi }^{+}\right)}^{2}+{\left(\eta_{\phi }^{+}\right)}^{2}$$

and

3.7 $$\kappa =\frac{\xi_{\phi }^{+}\eta_{\phi \phi }^{+}-\xi_{\phi \phi }^{+}\eta_{\phi }^{+}}{{\left(J^{+}\right)}^{3/2}}.$$

Finally, the dynamic boundary condition on the interface takes the form

3.8 $$\frac{F^{2}}{2}\left[\frac{1-Rf_{\phi }^{2}}{{\left(\xi_{\phi }^{-}\right)}^{2}+{\left(\eta_{\phi }^{-}\right)}^{2}}-(1-R)\right]+(1-R)\eta^{-}=0.$$

We seek solutions, symmetric about the centre of the wave, of the system (3.2)–(3.8) by a finite difference method. The numerical scheme for computing fully localized solitary waves is similar to [14], but modified for the present problem. The numerical computations are performed in a truncated long domain with length *L*. We introduce a uniform mesh

3.9 $$\phi_{i}=(i-1)\frac{L}{2N}-\frac{L}{2},\;i=1,2,\ldots ,N+1$$

and the corresponding unknowns

3.10 $$\left(\frac{d\eta^{+}}{d\phi }\right)(\phi_{i}),\;\left(\frac{d\eta^{-}}{d\phi }\right)(\phi_{i}),\;\left(\frac{d\kappa }{d\phi }\right)(\phi_{i}).$$

Therefore, $\xi_{\phi }^{\pm }$ and *f*_*ϕ*_ are the functions of $\eta_{\phi }^{\pm }$, and, briefly speaking, they can be computed in the Newton iteration algorithm in the following order:

$$\begin{array}{c} \text{computing }\xi_{\phi }^{-}\text{ via }(\text{3.2}) \end{array}⟹\begin{array}{c} \text{computing }f_{\phi }\text{ via }(\text{3.8}) \end{array}⟹\begin{array}{c} \text{computing }\xi_{\phi }^{+}\text{ via }(\text{3.4}) \end{array}$$

We calculate *η*^±^, *κ* and *f* by integrating their derivatives using the trapezoidal rule. The derivatives *η*^+^_*ϕϕ*_, $\xi_{\phi \phi }^{+}$ and *κ*_*ϕϕ*_ are computed by the five-point centred difference scheme. In fact, there are 3*N* unknowns, because $\eta_{\phi }^{+}(\phi_{N+1})=\eta_{\phi }^{-}(\phi_{N+1})=\kappa_{\phi }(\phi_{N+1})=0$ owing to the symmetry of the wave profiles. We then substitute all the expressions in (3.3), (3.5) and (3.7) evaluated at middle points $\phi_{i}^{m}≜(\phi_{i}+\phi_{i+1})/2$, *i*=1,2,…,*N* (a fourth-order interpolation formula is applied between the grid points and the middle points where necessary). This leads to 3*N* nonlinear algebraic equations for the 3*N* unknowns, which gives a closed system when the Froude number is given *a priori*.

The nonlinear system is solved via a Newton iteration algorithm, and the program is considered to have converged to a solution when the $L^{\infty }$ norm of the residual error is less than 10^−10^. The initial guess for Newton iteration is obtained by using the vanishing pressure method (see [24] for an example): an artificial fully localized pressure is applied either on the interface or on the free surface, then gradually eliminated using a continuation method by increasing the amplitude of the interface or free surface, and finally one obtains a solution without forcing. Once one solution is found, other solitary waves on the branch can be computed via a straightforward continuation method by changing the Froude number, density ratio, depth ratio or flexural rigidity as the parameter. Most of the computations were performed with the domain size *L* between 40 and 120, and the mesh size less than 0.1 to obtain sufficiently accurate numerical solutions.

As *E*_b_=0, the governing equations (2.2)–(2.8) reduce to those of pure gravity interfacial waves with a free surface. In this case, barotropic elevation solitary waves have been numerically computed for the full Euler system by Moni & King [11] and Woolfenden & Părău [14]. To validate our numerical code, we checked the results produced by our program against the results of these authors. For *R*=0.3, *H*=1 and *F*=1.404, Moni & King found the amplitudes of the interface and the free surface to be 0.134 and 1.552, whereas Woolfenden & Părău found them to be 0.137 and 1.562, respectively. Our numerical results read 0.137 and 1.562, which are in excellent agreement with those of Woolfenden & Părău, confirming the accuracy of our numerical method.

#### (ii) Fast (barotropic) solitary waves

As shown in §2*b*, the dispersion curve of *F*^+^ for the flexural–gravity problem always has a minimum at a non-zero wavenumber. Therefore, one can expect solitary waves with oscillatory decaying tails that bifurcate from infinitesimal periodic waves at the minimum of the external modes. It is noted that wavepacket barotropic solitary waves also appear in [14] when the term owing to elastic bending is replaced by surface tension.

**Figure 4.**
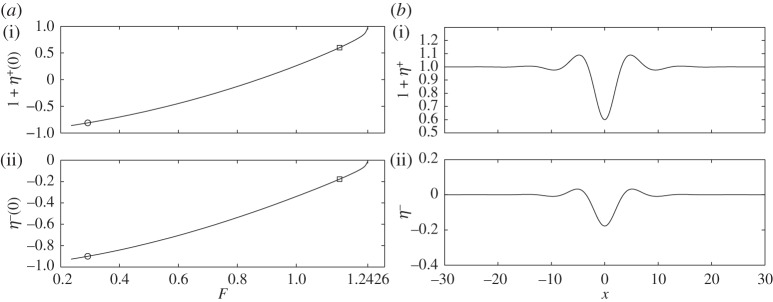
(*a*) Speed–amplitude bifurcation diagram for in-phase depression solitary waves with *H*=1, *E*_b_=1, *R*=0.9 and the bifurcation point *F*=1.2426 is predicted by linear theory (2.13); the upper branch is the free surface amplitude 1+*η*^+^(0), and the lower branch is the interfacial amplitude *η*^−^(0). (*b*) Typical wave profiles with *F*=1.1473, 1+*η*^+^(0)=0.6 and *η*^−^(0)=−0.1776 (points labelled by squares in *a*); note that the free surface (*a*(i),*b*(i)) and the interface (*a*(ii),*b*(ii)) are shown using different vertical scales.

**Figure 5.**
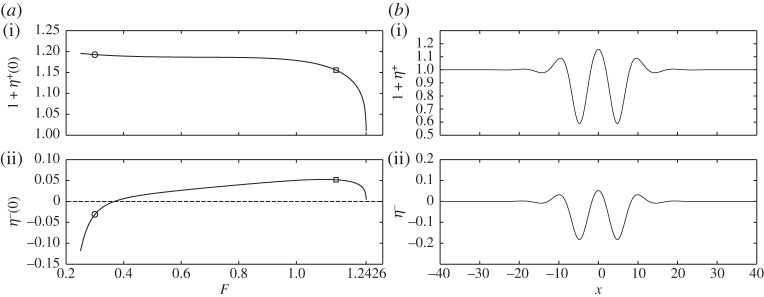
(*a*) Speed–amplitude bifurcation diagram for in-phase elevation solitary waves with *H*=1, *E*_b_=1, *R*=0.9 and the bifurcation point *F*=1.2426 is predicted by linear theory (2.9); the upper branch is the free surface amplitude 1+*η*^+^(0) and the lower branch is the interfacial amplitude *η*^−^(0). (*b*) Typical wave profiles with *F*=1.138, 1+*η*^+^(0)=1.1556 and *η*^−^(0)=0.0518 (points labelled by squares in *a*); note that the free surface (*a*(i),*b*(i)) and the interface (*a*(ii),*b*(ii)) are shown using different vertical scales.

**Figure 6.**
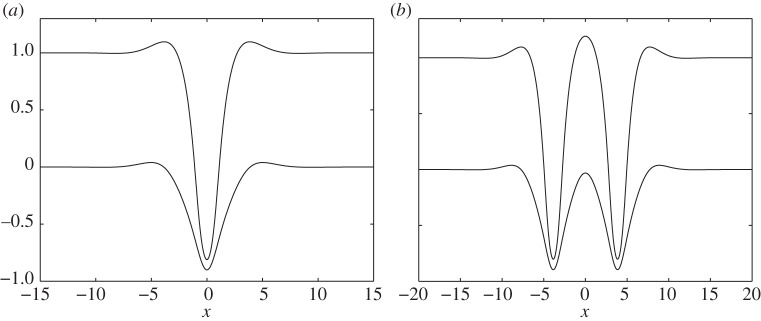
Highly nonlinear in-phase solitary wave profiles which are close to the extreme structure: (*a*) depression solitary waves (labelled by circles in figure 4*a*) with *F*=0.2918, 1+*η*^+^(0)=−0.81 and *η*^−^(0)=−0.9002 and (*b*) elevation solitary waves (labelled by circles in figure 5*a*) with *F*=0.3, 1+*η*^+^(0)=1.1927 and *η*^−^(0)=−0.0308.

Two branches of solitary waves are found: depression waves with troughs at *x*=0 and elevation waves with peaks at *x*=0. Figures 4*a* and 5*a* show the bifurcation diagrams for these two branches with *H*=1, *E*_b_=1 and *R*=0.9. The critical Froude number (i.e. the value of *F* corresponding to the minimum of the external mode) is then *F*^+^=1.2426. Two typical profiles for *F*=1.1473 and *F*=1.138 are shown in figures 4*b* and 5*b*. They correspond to depression and elevation waves, respectively. At very small amplitudes, it is well known that waves bifurcating from extrema of a single mode (i.e. one free boundary) dispersion relation at finite *k* approach wavepackets of monochromatic waves of critical *k* with envelopes described by the modulational cubic nonlinear Schrödinger equation (NLS) [23]. Rigorous justifications of the NLS equations exist for single layer fluids [25]. When a free surface and an interface are both present, the NLS analysis is much more complicated and beyond the scope of this paper. By following the bifurcation branches to the highly nonlinear regime, we found that the waves ultimately approach configurations where the free surface and the interface tend to touch each other. Figure 6 shows typical profiles which have developed remarkable, almost touching structures for both depression (*F*=0.2918) and elevation (*F*=0.3) solitary waves. We also have conducted similar computations by fixing *R*=0.9 and changing *H* and *E*_b_ by using a straightforward continuation method from the solutions shown. We found that the wave profiles and the bifurcation mechanism are qualitatively similar to those presented in figures 4–6.

The limiting configurations of figure 6 differ from those found for one-layer gravity–capillary or gravity–flexural solitary waves, which ultimately converge to overhanging structures with intersection points for the depression branch [24,26] or exhibit snake-like bifurcation behaviour connecting to multi-packet wave profiles for the elevation branch [27,28].

#### (iii) Slow (baroclinic) solitary waves

We now show that there is another type of solitary wave (baroclinic solitary waves) which bifurcate from the trivial uniform stream at values of the parameters corresponding to the maxima in figure 2*b*. Unlike the external mode *F*^+^, there are two possibilities for the extreme value (global maximum) of *F*^−^: it can be attained at zero wavenumber or at non-zero wavenumber (as shown in figure 2*b*). The properties of the out-of-phase solitary waves for these two cases are significantly different.

**Figure 7.**
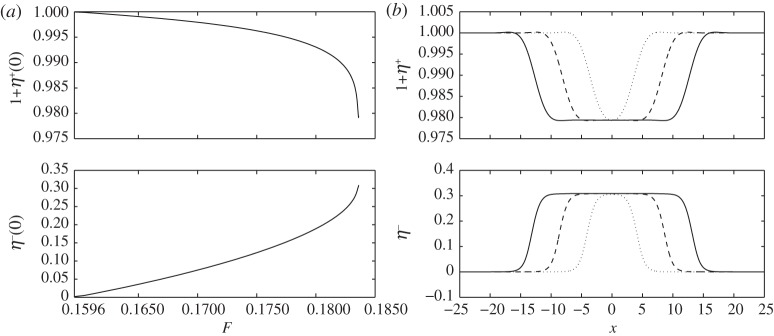
(*a*) Speed–amplitude bifurcation diagram for out-of-phase solitary waves with $H=\frac{1}{3}$, *E*_b_=0.5, *R*=0.9 and the bifurcation point *F*=0.1596 is the minimum of *F*^−^ of the linear theory; the upper branch is the free surface amplitude 1+*η*^+^(0) and the lower branch is the interfacial amplitude *η*^−^(0). (*b*) The typical wave profiles and the broadening as the Froude number increases: *F*=0.183594 (dotted line), *F*=0.183606 (dashed line) and *F*=0.183608 (solid line); the free surface profiles are shown at the top, whereas the interface profiles are shown at the bottom.

When the global maximum of *F*^−^ is attained at zero wavenumber, the bifurcation mechanism of the solitary waves is similar to those for interfacial long waves which, at small amplitude, are described by solutions of the Korteweg–de Vries equation. The waves are similar to the pure gravity interfacial solitary waves under the ‘rigid lid’ approximation [6,29], because the amplitude of the interface is relatively large compared with the displacement of the free surface. Figure 7 presents bifurcation diagrams and typical profiles of the solitary waves with a set of parameters $H=\frac{1}{3}$, *R*=0.9, *E*_b_=0.5. These waves bifurcate from the uniform stream line at *F*=0.1596 which coincides with the value predicted by the linear theory at *k*=0, and the amplitude of the wave increases as the Froude number increases as shown in figure 7*a*. The waves are of baroclinic type (free surface and interface have different phases) with a positive amplitude on the interface. The most conspicuous feature is a broadening of the wave, namely the midsections of the interface and the free surface develop plateaus, when the Froude number approaches a limiting value. The plateaus can become infinitely long; therefore, the flow in the far field and the flow in the middle can be referred to as parallel conjugate flows [30,29]. From this point of view, we can calculate the limiting value of *F* by solving the following algebraic equation:

3.11 $$(8+R)F^{6}-12(1+H)F^{4}+2(3-R){\left(1+H\right)}^{2}F^{2}-(1-R){\left(1+H\right)}^{3}=0.$$

This relation was first given by Dias & Il'ichev, and readers are referred to [16] for the detailed derivation (they derived (3.11) in the absence of an elastic cover, but their result remains valid for our problem, because the elastic sheet does not affect the result when the free surface and the interface are flat). The theoretic limiting value given by (3.11) is 0.1849, which is in agreement with the numerical result (≈0.1836) as shown in figure 7*a*. It is worth mentioning that, in the broadening process, both the amplitude and the Froude number change slowly approaching the respective limiting values. This fact indicates that neither of them is an appropriate parameter in the continuation method, and, therefore, in numerical computations, we use the area beneath the interface as the variable parameter as suggested by Turner & Vanden-Broeck [6].

**Figure 8.**
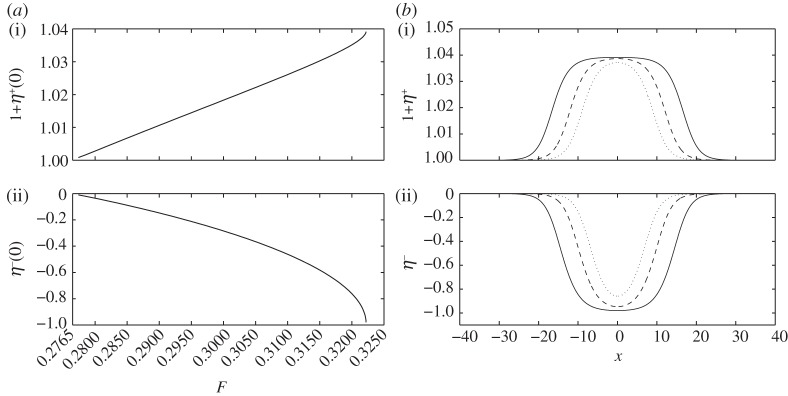
(*a*) Speed–amplitude bifurcation diagram for out-of-phase solitary waves with *H*=3, *E*_b_=0.1, *R*=0.9 and the bifurcation point *F*=0.277 is the minimum of *F*^−^ calculated by (2.14); the upper branch is the free surface amplitude 1+*η*^+^(0), and the lower branch is the interfacial amplitude *η*^−^(0). (*b*) The broadening of the wave profile as the Froude number increases: *F*=0.3215 (dotted line), *F*=0.322140 (dashed line) and *F*=0.322204 (solid line); the free surface (*a*(i),*b*(i)) and the interface (*a*(ii),*b*(ii)) are shown separately using different vertical scales.

Long interfacial waves with a rigid lid may be of elevation or depression depending on whether the equilibrium interface is above or below a particular depth (the midline in the limit as the densities are close; [29]). Thus, when we increase the relative depth *H*, the interfacial amplitude becomes negative, whereas the free surface and the interface are still in opposite phases. In figure 8*a,* we pick *H*=3, *R*=0.9 and *E*_b_=0.1, and plot the speed–amplitude bifurcation diagrams. The out-of-phase solitary waves under this set of parameters also display the broadening phenomenon (see the typical wave profiles in figure 8*b*). The numerical limiting value of *F* is about 0.3222, which coincides with the theoretical prediction (≈0.3203) calculated by (3.11).

We note that the elastic bending has little effect in the computation of the long solitary waves given the small variation of the free surface: we tested the profiles of the solitary waves under the set of parameters *H*=3, *R*=0.75 and *F*=0.51 by varying *E*_b_. It turns out that the wave profiles do not change appreciably, with the absolute difference between two profiles computed with different *E*_b_ being less than 10^−3^ for *E*_b_ varying in the range [0.01,1]. This is to be contrasted with the long solitary waves of the external mode [12,13]. There, because the elastic bending dramatically changes the dispersion curve of the external mode for large wavenumbers, non-decaying periodic tails are ruled out in the far field of long waves.

**Figure 9.**
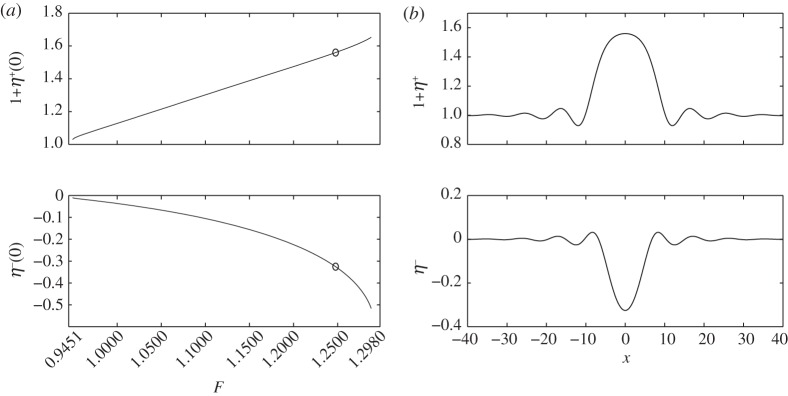
(*a*) Speed–amplitude bifurcation diagram for out-of-phase solitary waves with *H*=3, *E*_b_=2, *R*=0.1 together with two bifurcation points *F*=0.9451—the maximum of *F*^−^—and *F*=1.2980—the minimum of *F*^+^; the upper branch is the free surface amplitude 1+*η*^+^(0), and the lower branch is the interfacial amplitude *η*^−^(0). (*b*) The typical wave profile which is labelled by circles in (*a*): *F*=1.2477, 1+*η*^+^(0)=1.56 and *η*^−^(0)=−0.3257.

For the case when the maximum of the internal mode appears at a non-zero wavenumber, the bifurcation mechanism and the wave profiles are completely different from those mentioned above, in this section, and are similar to those mentioned in the previous section bifurcating from the minimum of the external mode. Consider the numerical experiment in figure 9. This experiment is carried out with *H*=3, *R*=0.1 and *E*_b_=2, and therefore the maximum 0.9451 of *F*^−^ occurs at *k*=0.7052. Typical wave profiles are shown in figure 9*b* which are wavepacket solitary waves featuring damped oscillatory tails. From the speed–amplitude bifurcation diagrams presented in figure 9*a*, we observe that the amplitude of this type of solitary wave increases as the Froude number increases, and that the branch fills the whole gap of speeds between the maximum of the internal mode and the minimum of the external mode. These solitary waves do not broaden, and thus the theoretical prediction of the limiting value of the Froude number (1.1547 under those parameters) is not applicable. It is to be noted that *no* in-phase solitary waves, which would bifurcate from infinitesimal periodic waves at the minimum of *F*^+^, are found in our numerical experiments under these parameters.

### (b) Generalized solitary waves

#### (iv) Series truncation method

Numerically, generalized solitary waves are computed via the modified version of the method stated in [13]. We approximate a generalized solitary wave by long periodic waves with period *L*. As done in §3*a*(i), the fluid geometry is mapped to the *ϕ*^±^−*ψ* domain with the upper layer [−*L*/2,*L*/2]×[0,*Q*^+^] and the lower layer [−*L*/2,*L*/2]×[0,−*Q*^−^], where *Q*^±^ are two unknown constants corresponding to the values of *ψ* on the free surface and the flat bottom. All the solutions can be approximated using truncated Fourier series, namely in the lower layer

3.12 $$x^{-}=\phi^{-}+\sum_{n=1}^{N}a_{n}\sin\left(\frac{2\pi n}{L}\phi^{-}\right)\left[\tanh\left(\frac{2\pi nQ^{-}}{L}\right)\sinh\left(\frac{2\pi n}{L}\psi \right)+\cosh\left(\frac{2\pi n}{L}\psi \right)\right]$$

and

3.13 $$y^{-}=\psi +a_{0}+\sum_{n=1}^{N}a_{n}\cos\left(\frac{2\pi n}{L}\phi^{-}\right)\left[\tanh\left(\frac{2\pi nQ^{-}}{L}\right)\cosh\left(\frac{2\pi n}{L}\psi \right)+\sinh\left(\frac{2\pi n}{L}\psi \right)\right]$$

and in the upper layer

3.14 $$x^{+}=\phi^{+}+\sum_{n=1}^{N}\sin\left(\frac{2\pi n}{L}\phi^{+}\right)\left[b_{n}\cosh\left(\frac{2\pi n}{L}\psi \right)+d_{n}\sinh\left(\frac{2\pi n}{L}\psi \right)\right]$$

and

3.15 $$y^{+}=\psi +b_{0}+\sum_{n=1}^{N}\cos\left(\frac{2\pi n}{L}\phi^{+}\right)\left[b_{n}\sinh\left(\frac{2\pi n}{L}\psi \right)+d_{n}\cosh\left(\frac{2\pi n}{L}\psi \right)\right].$$

Because there is a potential jump across the interface, we express *ϕ*^±^ as

3.16 $$\phi^{-}=\xi -s,\;\phi^{+}=\xi +s,\;\text{with}\;s(\xi )=\sum_{n=1}^{N-1}c_{n}\sin\left(\frac{2\pi n}{L}\xi \right).$$

Therefore, all the unknowns are parametrized by the variable *ξ*. It is obvious that, as *ψ*=−*Q*^−^, one has *y*^−^=−*Q*^−^+*a*_0_=−*H*, which gives *Q*^−^. Overall, we have the following 4*N*+4 unknowns: *a*_*n*_(*N*+1), *b*_*n*_(*N*+1), *c*_*n*_(*N*−1), *d*_*n*_(*N*), *B*^±^ and *Q*^+^. Accordingly, using the mesh (3.9), one can have *N*−1 equations from *x*^−^=*x*^+^ at the interface, *N*+1 equations from *y*^−^=*y*^+^ at the interface, 2*N*+2 equations from the dynamic boundary conditions on the interface and free surface. Additionally, two mean-value conditions complete the problem,

3.17 $$\int_{-L/2}^{L/2}y^{+}(\phi^{+},Q^{+})\phi_{\xi }^{+}\;d\xi =1,\;\int_{-L/2}^{L/2}y^{+}(\phi^{+},0)\phi_{\xi }^{+}\;d\xi =0.$$

We should remark that this series truncation method is not adequate to compute large-amplitude waves for our problem. As pointed out in [13], the computation of large-amplitude waves requires a large amount of mesh points, and, using this numerical scheme, the terms cosh⁡(2πnψ/L) and sinh⁡(2πnψ/L) in *x*^+^ and *y*^+^ grow exponentially as *n* increases. Thus, in §3*b*(ii), we focus mainly on relatively small-amplitude generalized solitary waves, and their complicated tails owing to the effect of elastic bending.

#### (v) Some numerical results

A generalized solitary wave consists of a localized midsection and non-decaying oscillatory tails. This type of wave arises from the scenario that a long wave [17,12,13,31] or a finite-amplitude wavepacket solitary wave [32] resonates with other linear waves which propagate at the same speed. Here, we consider the parameter regime in which long waves bifurcating from *F*^−^ at *k*=0 resonate with finite wavenumber waves from the external branch near *F*^+^. In order for this to happen before the internal waves broaden and reach a limiting amplitude, the gap between the maximum of *F*^−^ and the minimum of *F*^+^ must be sufficiently small (or non-existent). We note that this requires parameters that are unphysical for oceanic applications.

**Figure 10.**
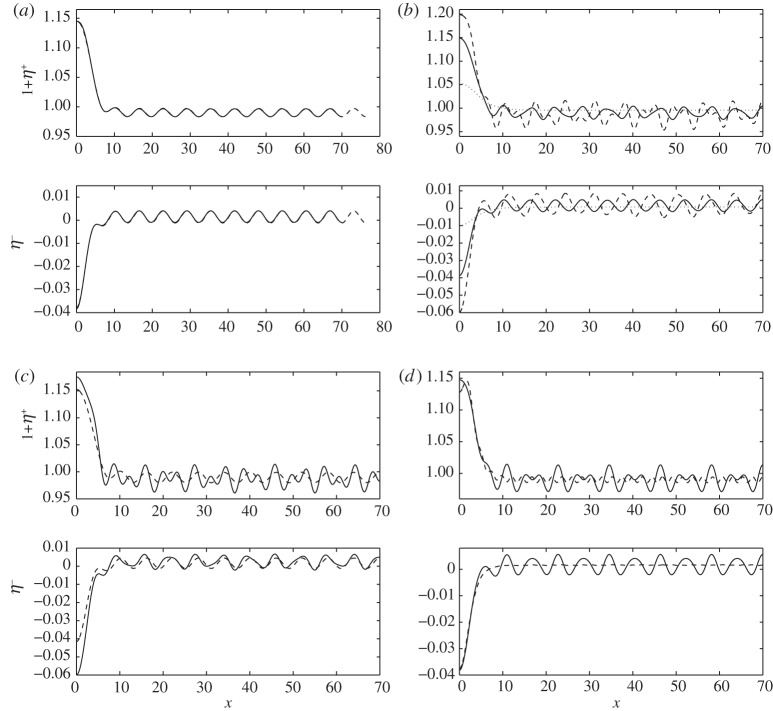
(*a*) Typical profiles of long periodic waves with non-decaying periodic tails with parameters *R*=0.1, *H*=3, *E*_b_=0.02 and *F*=1; superposition of the waves computed in different domains (*L*/2=70 for the solid line and *L*/2=76.3 for the dashed line) suggests the existence of the generalized solitary waves as L→∞. (*b*) Profiles of long free-surface and interfacial internal waves for *R*=0.1, *E*_b_=0.1, *H*=3, *L*/2=70 and three values of the Froude number: *F*=0.95 (dotted line), 1.00 (solid line), 1.03 (dashed line). (*c*) Profiles of generalized solitary waves for *R*=0.1, *E*_b_=0.1, *F*=1, *L*/2=70 and two different depth ratios: *H*=0.22 (solid line), *H*=0.28 (dashed line). (*d*) Profiles of the free surface and the interface for *R*=0.1, *H*=3, *F*=1, *L*/2=70 and two flexural–rigidity constants: *E*_b_=0.11 (solid line), *E*_b_=0.033 (dashed line).

Generalized solitary waves, which mathematically extend to infinity, are approximated by long periodic waves in the numerical experiments. As an example, we fix *R*=0.1 and first compute approximate long solitary waves as periodic waves with long flat troughs. Then, we vary the parameters *H*, *F* and *E*_b_ to modify the dispersion relation until a resonance occurs resulting in the appearance of periodic tails. For example, we first compute the wave with *R*=0.1, *H*=3, *E*_b_=0.1, *L*/2=70 and choose the Froude number *F*=0.95 which is in the gap between the minimum of *F*^+^ and the maximum of *F*^−^ in the dispersion relation. The dotted line in figure 10*b* shows that, for this Froude number, the wave is solitary-like and flat in the far field. We then increase the Froude number and ripples appear on the tails of the profiles as expected (see the dashed and solid lines in figure 10*b*) when *F* exceeds the minimum of *F*^+^.

In figure 10*a,* we present typical free-surface profiles and corresponding interfaces for the parameters *R*=0.1, *H*=3, *F*=1, *E*_b_=0.02, and the superposition of waves computed within two different domains. The midsection of the free surface and that of the interface are in opposite phases. It shows that, as the domain size increases, more and more periodic waves can be added in the far field without a notable change of the portion of the profile already obtained for smaller domain sizes. The computation strongly suggests the existence of generalized solitary waves in the limit as the size of the domain approaches infinity. Figure 10*d* demonstrates the formation of a trough at the origin of the free surface as the flexural–rigidity constant varies, indicating that curvature at the origin may change sign along with the changing parameters. There are many different families of generalized solitary waves characterized by an infinitely long train of ripples in the far field. The periodic tails of generalized solitary waves in a two-layer system with elastic cover are more complicated than that of the waves without an elastic sheet (i.e. *E*_b_=0). The solid lines in figure 10*c*,*d* show a Wilton-ripple-like periodic tail rather than the sinusoid-like tail of the pure gravity case. That is because *F*^+^ is not monotonic, and the finite-amplitude solitary waves can resonate with two linear periodic waves when the Froude number is above the minimum of *F*^−^. Generalized solitary waves with Wilton ripple far fields do not appear to have been calculated before. We conjecture that a similar phenomenon can also be observed in a two-layer system with a free surface, when the surface tension or interfacial tension is taken into account.

## 4. Conclusions

In a two-layer fluid system with a free upper surface, there are two distinct dispersion curves, *F*^+^ for the external mode and *F*^−^ for the internal mode. Depending on the specific physical effects, either on the free surface or on the interface, there may be a gap between *F*^+^ and *F*^−^ where varieties of gap solitary waves can exist. In this paper, we considered interfacial gravity waves with an elastic cover on the top of the upper layer fluid—a problem of interest in oceanic applications [4]. The nonlinear elastic model is based on the special Cosserat theory of hyperelastic shells. Steady, symmetric gap solitary waves are computed via a boundary integral method. For the realistic density ratios, there are two types of fully localized travelling waves: barotropic and baroclinic solitary waves. Barotropic solitary waves behave like one-layer free surface waves with wavepacket solitary waves, including depression and elevation waves, existing in this case. Both these are found to approach a limiting configuration that the free surface and the interface tend to touch each other. Baroclinic solitary waves, bifurcating from infinitesimal internal long periodic waves at Froude number *F*^−^(0), are restricted by a critical Froude number corresponding to parallel conjugate flows. If this critical Froude number is between the minimum of *F*^+^ and *F*^−^(0), the midsections of the interface and the free surface develop plateaus which become wider as the Froude number approaches its critical value from below. Baroclinic wave-packet solitary waves, bifurcating from finite wavenumber, are found for smaller density ratios when the maximum of *F*^−^ may occur at finite wavenumber. These solitary waves with decaying oscillatory tails are found numerically to be no longer confined by the critical Froude number, and can fill the entire gap between the minimum of *F*^+^ and the maximum of *F*^−^.

Small-amplitude generalized solitary waves are computed using a series truncation method and for the small density ratio. The Wilton-ripple-like periodic tails owing to the non-monotonicity of the external mode *F*^+^, which are distinct from the case when flexural effects are ignored, are highlighted. While it is beyond the scope of this paper, the computation of large-amplitude generalized solitary waves for the irrotational Euler system demands more refined numerical schemes and could be particularly interesting.

The stability of steady interfacial waves with free surface is an open problem and merits further investigation. The problem is complicated by the fact that interfacial waves induce a jump in tangential velocity across the interface resulting in small-scale shear instabilities. Despite this, we expect that waves similar to those we computed could be found in oceanic conditions or laboratory experiments as is the case of interfacial waves with a free surface in the presence of gravity alone. Furthermore, the dead-water phenomenon is likely to be observed when the load on the elastic sheet moves slowly enough to excite the internal modes.
